# Continuous-Wave Stimulated Emission Depletion Microscope for Imaging Actin Cytoskeleton in Fixed and Live Cells

**DOI:** 10.3390/s150924178

**Published:** 2015-09-18

**Authors:** Bhanu Neupane, Tao Jin, Liliana F. Mellor, Elizabeth G. Loboa, Frances S. Ligler, Gufeng Wang

**Affiliations:** 1 Department of Biomedical Engineering, University of North Carolina at Chapel Hill and North Carolina State University, Raleigh, NC 27695, USA; E-Mails: newbhanu@gmail.com (B.N.); lfmellor@ncsu.edu (L.F.M.); egloboa@ncsu.edu (E.G.L.); fsligler@ncsu.edu (F.S.L.); 2 Department of Chemistry, North Carolina State University, Raleigh, NC 27695, USA; E-Mail: tjin2@ncsu.edu

**Keywords:** super resolution optical microscopy, stimulated emission depletion (STED) microscopy, actin cytoskeleton, PC-12 cells, rat chondrosarcoma cells

## Abstract

Stimulated emission depletion (STED) microscopy provides a new opportunity to study fine sub-cellular structures and highly dynamic cellular processes, which are challenging to observe using conventional optical microscopy. Using actin as an example, we explored the feasibility of using a continuous wave (CW)-STED microscope to study the fine structure and dynamics in fixed and live cells. Actin plays an important role in cellular processes, whose functioning involves dynamic formation and reorganization of fine structures of actin filaments. Frequently used confocal fluorescence and STED microscopy dyes were employed to image fixed PC-12 cells (dyed with phalloidin- fluorescein isothiocyante) and live rat chondrosarcoma cells (RCS) transfected with actin-green fluorescent protein (GFP). Compared to conventional confocal fluorescence microscopy, CW-STED microscopy shows improved spatial resolution in both fixed and live cells. We were able to monitor cell morphology changes continuously; however, the number of repetitive analyses were limited primarily by the dyes used in these experiments and could be improved with the use of dyes less susceptible to photobleaching. In conclusion, CW-STED may disclose new information for biological systems with a proper characteristic length scale. The challenges of using CW-STED microscopy to study cell structures are discussed.

## 1. Introduction

In the past few years, with the help of fluorescence microscopy and various biotechnologies, visualization of sub-cellular structures and dynamic processes in fixed and live cells has become a routine procedure, with an associated accumulation of knowledge in biological structures and functions of biomolecules. However, further understanding of the biological structures and processes is often limited by current technologies, *i.e.*, the limited spatial resolution of optical microscopy. Electron microscopy has superior resolution; however, it cannot be used to probe dynamic cellular processes.

The advent of super resolution optical microscopy provides a new opportunity to study fine structures at the nanometer scale in live cells and tissue. To date, a number of microscopic approaches have been developed [1,2,3,4,5,6,7,8,9,10,11,12,13,14,15,16]. These methods include near-field scanning optical microscopy (NSOM) [5], stimulated emission depletion (STED) microscopy [6,7], stochastic optical reconstruction microscopy (STORM) [8,10] and photo activated localization microscopy (PALM) [9], structured illumination microscopy (SIM) [15,16], and other methods [11,12,13,14]. Among those techniques, STED microscopy has the advantages of combined high spatial resolution and temporal resolution as compared to single molecule localization-based microscopy or structured illumination microscopy. For example, the best reported lateral resolution for STED microscopy was ~6 nm for color centers in diamonds [17] while resolutions in the range of 20–150 nm were usually reported for biological samples. Video rate imaging is possible and was achieved at 28 frames per second (fps) with a lateral resolution of 62 nm for a field of view of ~2 μm × 2 μm [18]. In a recent variation of STED microscopy: REversible Saturable OpticaL Fluorescence Transitions (RESOLFT) microscopy, which uses photo-switching process to turn off unwanted fluorophores, as many as 100,000 focal plane donuts were used so that multiple pixels could be imaged simultaneously [19]. An imaging rate of ~2 fps was achieved on a 50 μm × 50 μm area with a lateral resolution of ~80 nm in live cell imaging with switchable, enhanced green fluorescent protein (GFP) to generate the fluorescent signal. In particular, high temporal resolution is of critical importance in imaging highly dynamic biological processes.

STED microscopes can be constructed using pulsed or continuous wave (CW)-laser sources [20,21]. In pulsed-STED microscopy, precise time delays are set between the excitation pulse, the depletion pulse, and the detection time window. The delayed depletion and gated detection can completely block unwanted scattering background and effectively reduce the depletion laser power/exposure time. As a result, pulsed-STED microscopy usually requires lower depletion laser power, uses a shorter depletion beam exposure time, and gives higher spatial resolution. However, the main disadvantage is the price. The total cost for a pulsed STED microscope currently is on the order of magnitude of ~1 million US dollars. The high quality pulsed lasers and the synchronization electronics are the main costs of the instrument and prices are unlikely to drop significantly in the near future. As an alternative, STED microscopes with CW laser sources can be built with much lower costs. We have constructed a CW-STED microscope with a budget of only ~200,000 US dollars [22]. With this CW-STED, we can achieve a resolution of 70 nm routinely on clear samples.

In this study, using actin as a challenging example, we explored the feasibility of employing a CW-STED microscope to study the fine structure of actins in fixed and live cells. Actin is a globular multi-functional protein that plays important roles in many cellular processes, including maintaining cell shapes [23,24], establishing cell junctions [25], cell movements [26], muscle contraction [27], cell division and cytokinesis [28], cell signaling [29], intracellular vesicle [30] and organelle movements [31], *etc*. Many of these processes involve highly dynamic (milliseconds to seconds time scale) formation and reorganization of fine structures of actin filaments and relevant cell organs [32]. Many diseases are associated with the malfunction of actin and/or its associated proteins [33]. Thus, it is important to understand how actin participates in cellular activities.

Actin structure and function has been studied extensively using conventional fluorescence microscopy and corresponding biotechnologies [34,35,36]. Recently, pulsed-STED microscopy has also been used in studying actin and new knowledge was obtained [37,38]. For example, using newly developed silicon-rhodamine (SiR) dyes, Lukinavicius *et al.* were able to observe the actin ring-like structures with a periodicity of ~180 nm at the rim of axons in primary rat hippocampal neurons [37], which is consistent with STORM results in fixed cells [39,40]. Urban *et al.* studied dynamic actin structures in dendrites and spines in live hippocampal neuron slices at various depths from the tissue surface at a frame rate of 11 s per frame (field of view: 20 × 20 μm) [38]. They found that chemical long-term potentiation induces a large number of spine necks to widen in neurons.

Here, we investigate the feasibility of using our CW-STED microscope to study actin structures and dynamics in fixed and live cells by comparing confocal and STED fluorescence microscopy. The homebuilt CW-STED system uses one of the most frequently used confocal fluorescence laser lines at 488 nm and corresponding confocal dyes fluorescein isothiocyante (FITC) and green fluorescent protein (GFP). We first optimized the conditions of STED imaging in cell environments. Then, we studied the actin structures in fixed PC-12 cells tagged with phalloidin-FITC and live chondrocyte cells expressing actin-GFP. Our results showed that the CW-STED microscope has improved spatial resolution compared to confocal microscopy and can be used to monitor changes in actin structures over time. Challenges include high scattering background and high bleaching rate. Nevertheless, CW-STED microscopy is a promising technique for studying fine cellular structures and dynamics in biological environments. It may disclose new information for systems with a proper characteristic length scale.

## 2. Experimental Section

### 2.1. STED Microscope Setup

**Figure 1:**
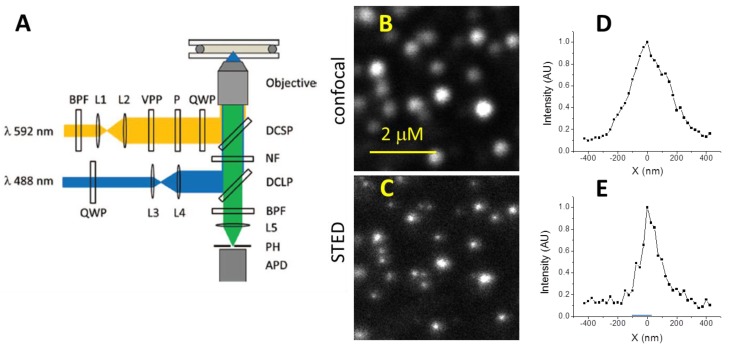
CW-STED microscopy. (**A**) Schematic of our home-built CW-STED microscope; (**B**) Confocal images of 45 nm FITC-doped polystyrene nanoparticles; (**C**) CW-STED image in the same area. The 2 µm scale bar applies to both images; (**D**) and (**E**) Representative cross-sections of the confocal and CW-STED images of the 45 nm particles.

The schematic of our home-built CW-STED microscope is shown in Figure 1A [22]. Briefly, we used a 488 nm laser line from an air-cooled Ar ion laser (35-LAP-431-240, CVI/Melles Griot) to provide excitation. The excitation laser beam was circularly polarized by a quarter-wave plate (QWP) (CVI/Melles Griot, ACWP-400-700-06-4) and expanded to overfill the back aperture of a microscope objective (Nikon, Plan Apo, 100×/1.40–0.7, Oil). A fiber laser (592 nm, 1.0 W, MPB communication, VFL-P-1000-592-OEM1) provided the depletion laser line. The depletion beam was expanded and passed through a 0–2 π vortex phase plate (RPC photonics, VPP1a) to generate a donut-shaped beam. The depletion beam was then cleaned with a Glan-type polarizer (Thorlabs), and circularly polarized by another QWP. The excitation and depletion beams were guided to the microscope objective back aperture by the combination of a 505 nm long-pass and a 570 nm short-pass dichroic mirrors. We used a 594 nm notch filter (Semrock, NF03-594-E) and a 535 ± 25 nm band-pass filter to remove the excitation and depletion laser light. The fluorescence signal was collected and imaged into a multimode optical fiber (Thorlabs) serving as a 50 µm (~0.8 AU) pinhole. The signal was detected by an avalanche photodiode (Perkin Elmer, SPCM-AQRH-15-FC) and counted with a computer board. A piezo-stage (PI Nano, Physik Instrumente, P-545, 1 nm precision) mounted on a manual XY translational stage was used for sample scanning in all XYZ directions. A home written macro program was used to synchronize the stage and the detector. During the image acquisition, the stage was scanned continuously. All presented STED images were collected with a size of 200 × 200 pixels irrespective of their physical sizes. The integration time was 1.0 ms/pixel unless specified otherwise. The acquired data were converted to images using a custom NIH ImageJ program.

In this study, we found that using a 200-mW depletion laser beam focused to the diffraction limited donut spot (corresponding to ~80 MW/cm^2^) provided a reasonable image quality while the photobleaching was lessened to an acceptable level. We were able to collect 5–10 images over the same area so that monitoring the actin structure change was possible over a limited time frame. All STED images presented were collected using a depletion beam power of 200 mW and an excitation beam power of 25 µW. In confocal imaging mode, the same microscope was used with the depletion laser beam turned off.

### 2.2. Cell Culture and Labeling of PC-12 Cells

PC-12 cells were purchased from ATCC (ATCC^®^ CRL-1721.1™). First, cells were grown in a collagen IV-coated 25 cm^2^ cell culture flask (BDE Biosciences) in a complete base medium (ATCC, RPMI-1640 medium) supplemented with 5% fetal bovine serum (ATCC^®^ 30-2020™)), 10% horse serum (ATCC^®^ 30-2040™)), and 1% penicillin streptomycin incubated under 37 °C, 95% humidity, and 5% CO_2_ conditions. After a week of growth in the culture flask, cells were subcultured in sterilized 2 cm^2^ petri dishes (Greiner Bio-One International) for 12 h on poly-L-lysine-coated microscope coverslips (Corning, 1.5 and 22 × 22 mm^2^). Then, cells were washed with 10 mL phosphate buffered saline (PBS) three times and fixed with 4% formaldehyde for 10 min. Next, cells were washed three times with phosphate buffer and incubated for six hours with a 10 μM solution of phalloidin (a bicyclic peptide isolated from the poisonous *Amanita phalloides* “death cap” mushroom) conjugated with green fluorescent dye fluorescein isothiocyante (FITC) (Molecular Probes^®^, F432). Finally, cells were washed with phosphate buffer three times to remove unbound phalloidin-FITC. The cells were imaged as described earlier in culturing medium and at room temperature (~25 °C).

### 2.3. Cell Culture and Transfection of RCS Cells

Rat chondrosarcoma (RCS) cells have been widely used for chondrocyte research, and the cell line used in this study was derived from the Swarm rat chondrosarcoma [41]. RCS cells were cultured in Dulbecco’s Modified Eagle Medium (Life technologies) containing 10% fetal bovine serum (FBS) (Premium Select, Atlanta Biologicals, Lawrenceville, GA, USA), 200 mM L-glutamine and 100 I.U. penicillin/100 µg/mL streptomycin (Mediatech, Herndon, VA, USA). The cells were cultured at 37 °C in 5% carbon dioxide until reaching 80% confluency, and then passaged using trypsin-EDTA (Invitrogen).

FuGENE 6 transfection kit (Promega) was used to transfect the actin-GFP plasmid into the RCS cells following manufacturer’s instructions. Briefly, 6 µg of plasmid DNA was mixed with FuGENE transfection reagents/medium and incubated at room temperature for 15 min. The FuGENE transfection reagent/DNA plasmid mixture was then added to 300,000 cells, and incubated overnight in a four-chamber slide (Fisher). Transfection efficiency was assessed using fluorescent microscopy and bright field images.

## 3. Results and Discussion

### 3.1. Resolution of STED Microscopy

The spatial resolution of STED microscopy is highly dependent on the depletion laser beam profile and intensity [22]. Using the full power of a 1-W fiber laser at 592 nm as the depletion source, we were able to achieve a full-width at half maximum (FWHM) spatial resolution of 70 nm routinely for our home-built system. However, we found that for biological samples, the depletion laser power needed to be reduced because: (1) biological cells contain a lot of particulates, which strongly scatter the laser light, deteriorating the image quality; and (2) photobleaching of the dye molecules becomes significant as the oxygen concentration is high in cytoplasm; thus the number of depletion laser exposures was limited to two or three.

For these reasons, we systematically studied the optimal conditions for CW-STED imaging in biological environments. Essentially, we lowered the depletion laser power while monitoring the image quality and spatial resolution for images of living cells. We found that by reducing the depletion laser power to ~200 mW (~80 MW/cm^2^), the photobleaching was reduced and ~10 high quality STED images could be obtained from the same area (detailed discussed in Section 3.3). The FWHM of the cross sections of 45 nm green fluorescent polystyrene nanoparticles (Invitrogen) in cell culture medium was 113 ± 15 nm measured from six particles in the image. Considering the convolution of the particle size and the STED point spread function (PSF), the actual FWHM resolution of our CW-STED microscope is 100 ± 15 nm at designated experimental condition. Figure 1B,C show the confocal and STED images of 45 nm FITC doped polystyrene nanoparticles. It is apparent that CW-STED microscopy improves the spatial resolution and resolves individual particles that show up as blobs using the same optics in a confocal microscopy mode. Under this condition, the FWHM spatial resolution was improved by a factor of ~3 (from 275 ± 25 nm for confocal to 100 ± 15 nm for STED microscopy).

### 3.2. CW-STED Imaging of Fixed PC-12 Cells

**Figure 2:**
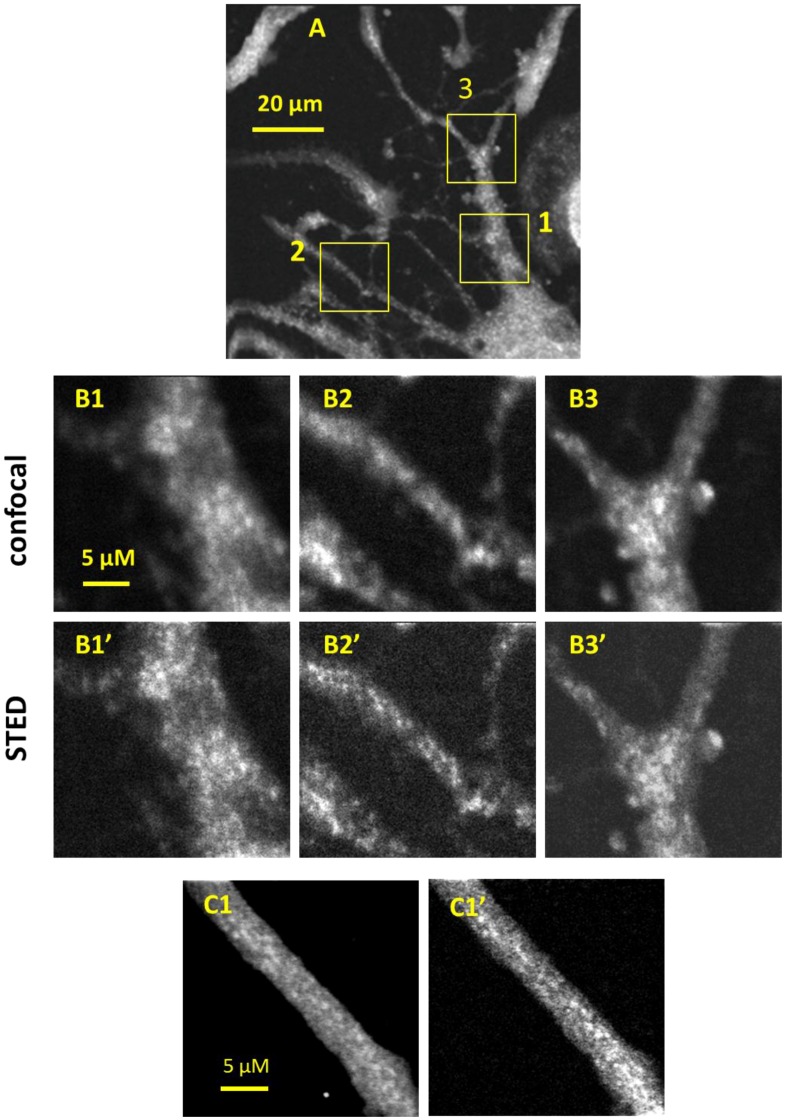
Confocal and CW-STED images of fixed PC-12 cells stained with phalloidin-FITC. (**A**) Confocal image of PC-12 cell with different sized neurites; (**B**) Expanded views of three selected neurites of different diameters; B and B’ refer to confocal and STED images, respectively; (**C**) A pair of confocal and STED images of another typical neurite showing an increase in geometric resolution for the STED image.

We used the microscope to study actin cytoskeleton in neurites of PC-12 cells using both the CW-STED and confocal configurations. The PC-12 cells were differentiated, fixed, and labeled with phalloidin-FITC. Phalloidin is a bicyclic polypeptide isolated from a poisonous species of mushroom and is known to label polymeric actin (F-actin) with very high specificity [42]. Figure 2A shows the differentiated PC-12 cells, and Figure 2B shows the confocal and STED images of expanded views of interest in Figure 2A. The STED images show much improved resolution and more structural details. Compared to the blurred images in the confocal micrograph, STED microscopy resolved multiple small bright spots, indicating that the big blobs in confocal images are composed of multiple actin filament bundles or aggregates. The FWHM of linear traces over these small bright features in the STED images ranged from 200 to 400 nm, suggesting the size of the actin filament aggregates. Due to the limit of the spatial resolution, we were unable to resolve individual actin filaments.

Especially of interest is that actin filaments seem to aggregate at the periphery of small neurites of the cells. This can be seen in the CW-STED images (e.g., Figure 2-B2’), while the confocal images only show a blurred neurite tube (Figure 2-B2). It is known from STORM microscopy that actin filaments form periodic ring-like structures with a spacing of ~190 nm in neuronal axons [39,40]. It is unclear how actin filaments are organized in neurites. Our data suggests that actin filaments may support the tubular structure in neurites in much the same manner as they do for axons. How they are organized to achieve this function needs to be further studied.

### 3.3. CW-STED Imaging of Live Chondrocytes

**Figure 3:**
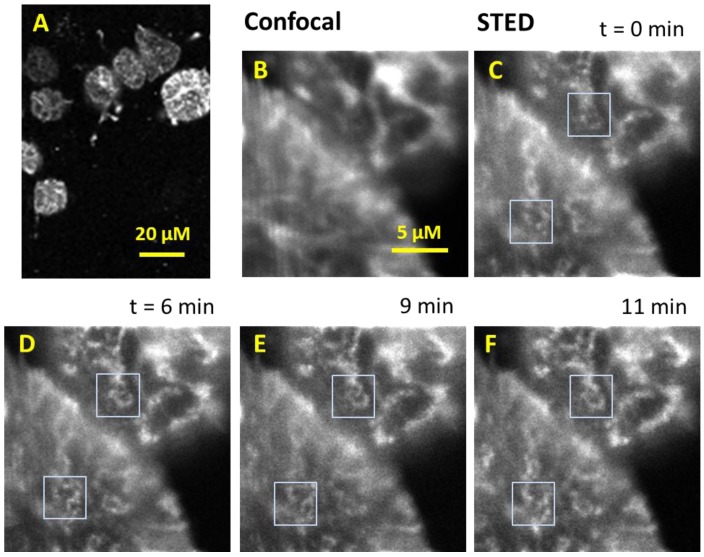
Confocal and STED images of live rat chondrocyte cells transfected with green fluorescent protein. (**A**) Confocal image of chondrocyte cells; (**B**) Confocal image of selected cells; (**C**–**F**) STED images of the same cells at different times. The scale bar in (B) also applies to (C–F).

We also tested the performance of our CW-STED microscope in imaging live rat chondrosarcoma cells expressing GFP-actin. Confocal fluorescence images show that actin forms bundled filaments at and near the cell periphery (Figure 3A). Figure 3B shows the confocal image of a small portion of two RSC cells. The corresponding STED image is shown in Figure 3C. Once again, STED images reveal many fine structures that are not disclosed by conventional confocal microscopes. Specifically, the CW-STED images show thinner actin filament bundles or smaller aggregates for otherwise smeared images in confocal microscopy. As a quantitative measure, we randomly picked 10 thin actin filaments and measured their width, which gave an average of 275 ± 50 nm. The measured width is the convolution of the actual filament width and the STED PSF. As a comparison, the widths of the same actin filaments in confocal images were measured to be 510 ± 60 nm.

We also took time lapse CW-STED images of the same area of the live chondrocytes. Figure 3D–F show the same area as in Figure 3B at 6, 9, 11 min after the first STED image. Compared to Figure 3C, we can observe that the actin filaments had significant fine structural changes as illustrated in blue frames, suggesting dynamic changes in the organization of the actin proteins. Note these structural changes are not evident using confocal fluorescence microscopies.

**Figure 4:**
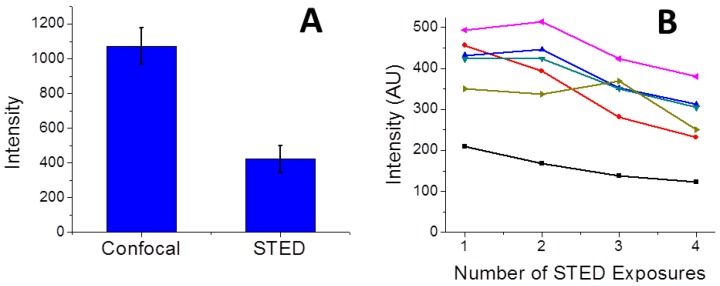
Fluorescence intensity in CW-STED imaging. (**A**) Comparison of confocal and STED image intensity. The intensity was measured from the maximum intensity of five cell features with similar intensities in Figure 3. The error bar is calculated from the standard deviation of the intensity of the five cell features; (**B**) Photobleaching of STED image upon multiple exposures to the depletion laser beam. Black curve: the average intensity of the whole image in Figure 3. The other five curves: the maximum intensity of five selected cell features in Figure 3 at different exposures. The total image intensity decays smoothly while the cell feature intensity fluctuated slightly because of the movement of the live cell.

Currently, we are using 1.0 ms integration time for each pixel in both confocal and STED imaging. With the 1-ms integration time, we were able to collect an image within seconds to 10 s of seconds depending on the size of the image. Thus, we are able to monitor actin morphology change using CW-STED microscopy with a temporal resolution of seconds. The STED image intensity was typically 30%–50% of that of the confocal images for the same feature in the live cells (e.g., 38% in Figure 4A). Thus, the imaging rate of STED microscopy was on the same order of magnitude as that of the embedded scanning confocal microscope, using the signal strength as the limiting factor for the imaging rate. This is consistent with literature reports. For example, Moneron *et al.* has achieved an imaging time below 0.2 s with a CW-STED microscope [20]. Even higher temporal resolution can be obtained using the more expensive pulsed STED microscopes [18,38]. The STED image intensity drops to 64% of the initial image intensity at the 4th exposure in the same series of measurements (Figure 4B). It is expected that photobleaching by the depletion laser beam would be the major hurdle for repeated observation of the same cells over extended periods of time.

### 3.4. Challenges and Opportunities

We have shown improved spatial resolution and more structural information about actin cytoskeleton in both fixed and live cells by using CW-STED microscopy. However, in the experiments, we had to sacrifice spatial resolution by reducing depletion laser power due to two reasons: (1) First, the light from the depletion laser is scattered by the sub-cellular structures. The scattered light increases the background and decreases the fluorescence signal resolution. The high scattering background is more problematic for CW-STED than in pulsed STED microscopy, where the scattering of the depletion laser beam can be gated out [7]; (2) Second, the powerful depletion laser beam photo-bleaches the dyes. The power of the depletion beam needs to be several orders of magnitude times stronger than that of the excitation beam. In the presence of oxygen, conventional fluorescent dyes are bleached quickly, decreasing image quality and irreversibly destroying the dyes. Further, we can only obtain a very limited number of collections in the same area, which prevents the continuous study of dynamic events. Two improvements would help address these issues: (1) Better dyes are needed that are more resistant to photobleaching. Note that in the literature, a variety of dyes with improved photo-stability such as Alexa Fluor 488 [20] and Tetramethylrhodamine (TMR) [43] have been reported and the search for more robust STED dyes, e.g., silicon-rhodamine (SiR) dyes, continues [37]; (2) Shifting the excitation and depletion to the red or even IR region may be advantageous because of the low photon power and low scattering tendency for the IR light; however, the resolution would be compromised due to the increase of the wavelength. If we can solve these issues, we will have a powerful tool to study the dynamics of the fine structure of actin filaments that is unparalleled with other current techniques.

## 4. Conclusions

In this study, we explored the feasibility of using a CW-STED microscope to study fine structure and dynamics of the actin cytoskeleton. We obtained improved spatial resolution of the actin filaments in both fixed PC-12 cells and live rat chondrosarcoma cells compared to confocal microscopy. We also demonstrated the ability to image changes in actin organization over time. These data show that CW-STED imaging is an affordable and promising technology to study dynamic sub-cellular features that are not resolvable under conventional optical microscopies. In recent years, many interesting examples of STED microscopy in cell and tissue level imaging have been reported [21]. To fully exploit the high spatial resolution of STED microscopy, we need to resolve the photobleaching issue because high power of the depletion laser power is currently a must in both CW- and pulsed STED microscopy. This issue may be solved by designing better STED dyes that are easily depleted and more resistant to photobleaching.
